# Fusogenicity of SARS-CoV-2 BA.2.86 subvariant and its sensitivity to the prokaryotic recombinant EK1 peptide

**DOI:** 10.1038/s41421-023-00631-2

**Published:** 2024-01-09

**Authors:** Lijue Wang, Fanke Jiao, Hanxiao Jiang, Yitao Yang, Ziqi Huang, Qian Wang, Wei Xu, Yun Zhu, Shuai Xia, Shibo Jiang, Lu Lu

**Affiliations:** 1 Key Laboratory of Medical Molecular Virology (MOE/NHC/CAMS), Shanghai Institute of Infectious Disease and Biosecurity, School of Basic Medical Sciences, Shanghai Frontiers Science Center of Pathogenic Microorganisms and Infection, Fudan University, Shanghai, China; 2 National Key Laboratory of Biomacromolecules, CAS Center for Excellence in Biomacromolecules, Institute of Biophysics, Chinese Academy of Sciences, Beijing, China

**Keywords:** Molecular biology, Cell biology

**Fig. 1:**
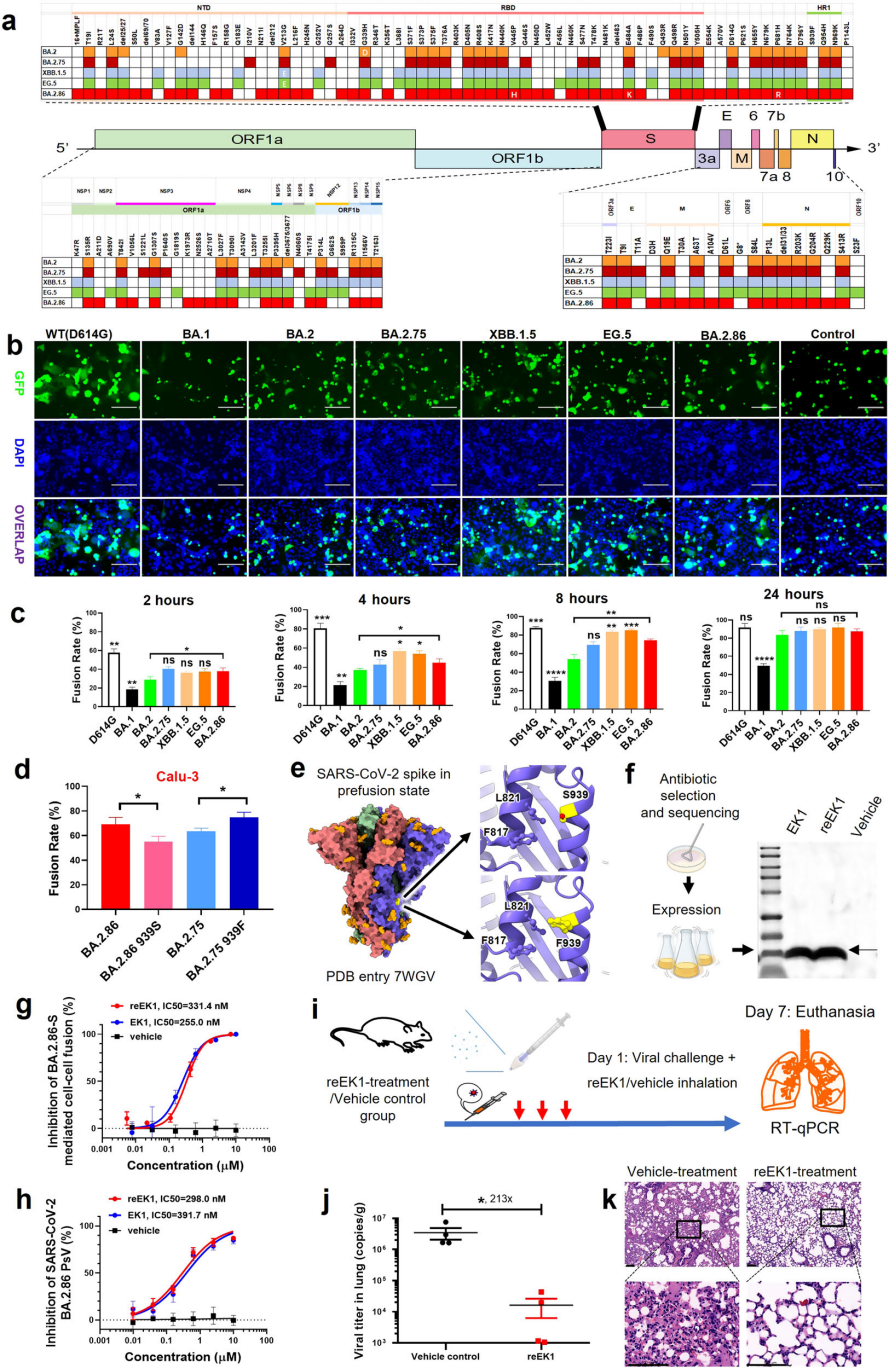
Fusogenicity of BA.2.86 S protein and the antiviral efficacy of reEK1. **a** Distinct S protein mutational profile of the Omicron subvariant BA.2.86. **b** Representative images of cell-cell fusion mediated by 293 T/WT(D614G)-S, BA.1-S, BA.2-S, BA.2.75-S, BA.2.86-S, XBB.1.5-S, EG.5-S, or negative control after coculture for 8 h. Scale bars, 150 µm. **c** Rate of fusion mediated by WT(D614G)-S, BA.1-S, BA.2-S, BA.2.75-S, BA.2.86-S, XBB.1.5-S and EG.5-S proteins on Calu-3 cells after coculture for 2, 4, 8 and 24 h. As compared with the BA.2.86 group, asterisks indicate significant differences (*P < 0.05; **P < 0.01; ***P < 0.001, ****P < 0.0001). **d** Alteration of fusion capacity mediated by S939F mutation in the HR1 region. **e** Effect of S939F mutation on the prefusion state of S protein. Surface representation displays the structure of WT S trimer (PDB entry 7WGV) with S939 residue highlighted in yellow. Predicted structure of the S939F mutant was obtained from the SWISS-MODEL server. Zoomed-in view depicts the local region surrounding S939 and F939, as represented in cartoon form. Crucial residues are depicted as sticks and labeled accordingly. **f** Schematic representation of prokaryotic expression system for production of reEK1 peptide. **g** Efficacy of reEK1 and EK1 peptides against BA.2.86-S-mediated cell–cell fusion. **h** Quantitative analysis of the inhibitory activity of reEK1 and EK1 peptides against BA.2.86 PsV infection. **i** Schematic representation of in vivo protective experiments of reEK1 through aerosolization inhalation route against BA.2 variant challenge in Tgtn (CAG-human ACE2-IRES-Luciferase) mouse model. **j** Quantitative analysis of viral titer reduction by reEK1 protection against authentic BA.2 variant in Tgtn (CAG-human ACE2-IRES-Luciferase) mouse model. **k** Protective efficacy of reEK1 against BA.2.86 infection that causes histopathological changes in mouse lung tissues. Scale bars, 100 µm.

Dear Editor,

BA.2.86, a novel SARS-CoV-2 Omicron subvariant, has emerged along with widespread concerns since the number of mutations in its S protein exceeds that of other Omicron subvariants (Fig. 1a). On August 18, 2023, the World Health Organization formally classified BA.2.86 as a variant under monitoring (VUM)^1^. Notably, numerous studies have reported that BA.2.86 has evaded humoral immunity induced by both inactivated and mRNA SARS-CoV-2 vaccines, as well as COVID-19 convalescent plasma^2,3^. However, in comparison to previous dominant SARS-CoV-2 Omicron subvariants, the fusogenicity of BA.2.86 and its sensitivity to coronavirus fusion and replication inhibitors have yet to be systematically evaluated.

Through analysis of the BA.2.86 genome, we discovered mutations with the potential to impact immune evasion, and infectivity (Fig. 1a). When compared to its ancestor BA.2, BA.2.86 was found to have 33 mutations in its S protein, including 28 substitutions, 4 deletions, and 1 insertion. This unique S-mutant profile sets BA.2.86 apart from other prevalent SARS-CoV-2 Omicron subvariants (Fig. 1a). In particular, P681R and S939F mutations located at the viral S1/S2 cleavage site and HR1 region, respectively, may impact viral fusogenicity, a crucial feature in determining the fusion capacity of SARS-CoV-2 variants and subvariants^4^.

Here, we systematically evaluated the fusion kinetics driven by BA.2.86 S protein, as well as S proteins of wild-type (WT)-D614G, BA.1, BA.2, BA.2.75, XBB.1.5 and EG.5, on human lung tissue-derived Calu-3 cells (Fig. 1b). As shown in Fig. 1c, BA.2.86-S mediated fusion rates of 37.9%, 44.82%, and 74.17% at the 2-, 4-, and 8-h timepoints, respectively, significantly higher than those mediated by BA.2-S, similar to those mediated by BA.2.75-S, but inferior to those mediated by XBB.1.5-S, EG.5-S and WT-S (D614G). At the 24-h timepoint, most Omicron subvariants exhibited a virus-mediated fusion rate exceeding 85%, whereas Omicron variant BA.1 only showed a fusion rate of about 50% (Fig. 1c). The result of weak fusogenicity mediated by BA.1-S is consistent with that from previous studies^5^. The efficiency of fusion among the human intestine-derived Caco-2 cells and 293T-ACE2 cells mediated by these Omicron subvariant S proteins exhibited similar trends (Supplementary Fig. S1a, b). Among these subvariants, BA.2.86-S drived an intermediate level of fusion kinetics, suggesting that this subvariant only harbors mild pathogenicity. Nonetheless, its expected evolution in the future still requires close attention.

Interestingly, compared with BA.1 and BA.2 S proteins, BA.2.86-S showed augmented S1/S2 cleavage in the biosynthesis process (Supplementary Fig. S1c, d). As noted above, we found the S939F mutation to be located at the HR1 domain. Therefore, we further analyzed its effect on the formation of six-helix bundle (6-HB) between HR1 and HR2, a critical core structure involved in viral and cell membrane fusion. As shown in Fig. 1d and Supplementary Fig. S2a, b, BA.2.86-S with the recovered F939S mutation (BA.2.86-S-F939S) showed decreased fusion capacity, compared to the increased fusogenicity of BA.2.86-S-S939F. Consistently, S939F could significantly strengthen the fusogenicity of BA.2.75-S, suggesting that the S939F mutation can potentiate viral fusion.

To further unravel the molecular mechanism underlying increased membrane fusion efficiency resulting from the S939F mutation, we performed a comparative analysis using the homologous modeling method to predict the structure of mutant SARS-CoV-2 S protein. In the prefusion state of S protein, the S939 residue was situated on the protein surface in proximity to two hydrophobic residues, L821 and F817 (Fig. 1e). In the WT S protein, S939 did not engage in any interactions with these two residues. However, the mutation of S939 into F939 leads to the formation of hydrophobic interactions with the neighboring L821 and F817. This interaction fortifies the affinity between the helixes harboring S939 and L821, thereby increasing the stability of S protein in the prefusion state. Importantly, however, this interaction toward S protein stability does not appear to rigidly lock the S protein to the prefusion conformation; instead, S protein only stably awaits receptor binding and the initiation of membrane fusion. Meanwhile, we explored the effect of the S939F mutation in BA.2.86-S on its S1/S2 cleavage efficiency in biosynthesis process. Interestingly, the western blot result indicated that BA.2.86-S-F939S bearing the recovered mutation F939S showed weaker S1/S2 cleavage compared to the original BA.2.86-S (Supplementary Fig. S2c, d). Consistently, the S1/S2 cleavage in BA.2.75 was enhanced after introducing S939F mutation (Supplementary Fig. S2c, d). Hence, the S939F mutation in BA.2.86-S can either improve the S1/S2 cleavage efficiency during its protein biosynthesis or significantly increase the stability of BA.2.86-S protein in the prefusion state, thus being doubly beneficial to viral fusogenicity.

Given the increased fusogenicity of BA.2.86-S accompanied by HR1-mutation, we further evaluated the efficacy of our pan-CoV fusion inhibitor, EK1, which specifically targets the viral HR1 region, and currently is in phase III clinical trials^6,7^. As expected, EK1 showed very potent fusion inhibitory activity against BA.2.86-S-mediated fusion with half-maximal inhibitory concentration (IC_50_) of 255 nM, showing the promise of EK1 as a candidate fusion inhibitor against BA.2.86 subvariant. To confirm this conclusion, we further predicted a homologous model of the HR1/EK1 complex with specificity toward S protein harboring the S939F mutation (Supplementary Fig. S3a). Since neither S939 nor F939 appears to account for the affinity of EK1 peptide toward HR1 trimer, it can be concluded that this mutation does not compromise the peptide’s efficacy.

In order to substantially reduce the production cost of the synthetic peptide-based drug, we developed a prokaryotic expression system to produce a recombinant EK1 peptide (reEK1) for prophylactic administration. To accomplish this, we fused the EK1 sequence to the C-terminus of the expression tag thioredoxin (TRX). After purification of the recombinant protein TRX-reEK1 obtained from the prokaryotic expression system, the TRX tag was removed through enzymatic cleavage using TEV Protease. This approach yielded the pure reEK1 peptide, the sequence of which is consistent with that of the chemically synthesized EK1 peptide. Remarkably, this expression methodology yielded an 80 mg/L reEK1 peptide from fermentation liquid within the confines of the laboratory, and the resultant peptide showed > 99% purity (Fig. 1f; Supplementary Fig. S3b–d). Moreover, this innovative technique could become significantly more cost-effective when it is scaled up for industrial production, offering distinct advantages over conventional chemical synthesis approaches. We found that reEK1 could also potently inhibit BA.2.86-S fusion with IC_50_ at 331 nM (Fig. 1g). Both reEK1 and EK1 showed potent inhibitory activity against BA.2.86-S pseudovirus (PsV) infection with IC_50_s of 298 and 392 nM, respectively (Fig. 1h). Additionally, reEK1 broadly inhibited infection of BA.2, BA.2.75, XBB.1.5 and EG.5 PsVs with IC_50_s in the range of 243 nM to 420 nM, which represents inhibitory potency comparable to that of synthetic EK1 peptide (Fig. 1h; Supplementary Fig. S4a, b).

To further assess the in vivo efficacy of reEK1, we evaluated the prophylactic efficacy of reEK1 using an hACE2-Tg mouse model challenged with authentic BA.2, as no authentic BA.2.86 was available. As shown in Fig. 1i, reEK1 administered through aerosolization inhalation route could significantly protect mice from authentic BA.2 variant infection. On the 7th day post-infection, viral titer in mouse lung tissue in reEK1 group was significantly reduced by 213-fold (Fig. 1j), compared with that of the vehicle control group. Notably, based on pathological examination, mice in the viral control group presented typical histopathology on the 7th day of post-infection, including alveolar septal thickening and inflammatory infiltrates in lung tissue, while mice in the reEK1-treatment group showed attenuated pathological changes in their lung tissue (Fig. 1k). Collectively, reEK1 exhibits ideal in vitro and in vivo antiviral efficacy.

Overall, we systematically studied the fusogenic kinetics of BA.2.86 S protein on human lung cells. Compared with parental BA.2-S, BA.2.86-S showed increased fusogenicity, albeit still inferior to that of XBB.1.5-S and EG.5-S, suggesting that BA.2.86 only possesses mild pathogenicity. In this study, we found that BA.2.86 mutation S939 specific to the HR1 region promoted BA.2.86-S fusion. Interestingly, based on structural analysis, we found that the S939F mutation could stabilize the prefusion state of BA.2.86 S protein, but had little effect on 6-HB formation in the post-fusion state, resulting in increased viral fusogenicity. To meet this challenge, we herein developed a prokaryotic expression platform for the production of recombinant (re)EK1, which is completely identical to the synthetic EK1 peptide, but with the advantages of high production and low cost. More importantly, reEK1 showed potent in vitro and in vivo antiviral activity against BA.2.86 and other dominant Omicron subvariants, suggesting that the prokaryotic reEK1 expression system could eventually replace synthetic EK1 for clinical use with attendant large-scale production and lower production costs.

### Supplementary information

Supplementary Information
