# Supplementary material for: Fusogenicity of SARS-CoV-2 BA.2.86 subvariant and its sensitivity to the prokaryotic recombinant EK1 peptide
Source: Cell Discov. 2024 Jan 9;10:6. doi: 10.1038/s41421-023-00631-2 (PMC10774434; doi:10.1038/s41421-023-00631-2)
Supplement: Supplementary file 1 — Supplementary Information [file 41421_2023_631_MOESM1_ESM.pdf]

Fusogenicity of SARS-CoV-2 BA.2.86 subvariant and its sensitivity to the prokaryotic recombinant EK1 peptide

Lijue Wang[1], Fanke Jiao[1], Hanxiao Jiang[1], Yitao Yang[1], Ziqi Huang[1], Qian Wang[1], Wei Xu[1], Yun Zhu[2,†], Shuai Xia[1,†], Shibo Jiang[1,†], Lu Lu[1,†]

Affiliations:

[1]Key Laboratory of Medical Molecular Virology (MOE/NHC/CAMS), Shanghai Institute of Infectious Disease and Biosecurity, School of Basic Medical Sciences, Shanghai Frontiers Science Center of Pathogenic Microorganisms and Infection, Fudan University, Shanghai, China.

[2]National Key Laboratory of Biomacromolecules, CAS Center for Excellence in Biomacromolecules, Institute of Biophysics, Chinese Academy of Sciences, Beijing 100101, China.

†Corresponding author. Email: lul@fudan.edu.cn (L.L.); shibojiang@fudan.edu.cn (S.J.); sxia15@fudan.edu.cn (S.X.); zhuyun@ibp.ac.cn (Y.Z.).

Methods

Cell lines, plasmids, and peptides

Calu-3 cell line and the Caco2 cell line were purchased from the Chinese Academy of Science Cell Bank (China); 293T cells and 293T/ACE2 cells were maintained in our laboratories. All cell lines were seeded in Dulbecco's Modified Eagle's Medium (DMEM) with 10% fetal bovine serum (FBS) in sterilized flasks. All plasmids were synthesized or stored in our laboratories, including pAAV-SARS-CoV-2-S (BA.2/ BA.2.75/ BA.2.86/ XBB.1.5/EG.5/D614G)-IRES-EGFP, pcDNA-3.1-SARS-CoV-2-S (BA.2/BA.2.75/ BA.2.86/ XBB.1.5/ EG.5/D614G), pNL4-3.Luc.R-E and pAAV-IRES-EGFP. *S* gene sequences of SARS-CoV-2 are obtained from GIASID, including: wild-type (WT)-D614G (EPI_ISL_412912), BA.2 (EPI_ISL_9022266), BA.2.75 (EPI_ISL_13583747), BA.2.86 (EPI_ISL_18138566), XBB.1.5 (EPI_ISL_16346200) and EG.5 (EPI_ISL_18052033).

The sequences of EK1, as previously described[1-3], was "SLDQINVTFLDLEYEMKKLEEAIKKLEESYIDLKEL". EK1 and reEK1 peptide powders are dissolved in DMEM.

Expression and purification of reEK1

Based on the amino acid sequence of the EK1 peptide, the corresponding nucleotide sequence was subjected to codon optimization for expression in the E. coli system and synthesized. The nucleotide was cloned into the pET-32a expression vector and introduced into the E. coli expression strain BL21 (DE3). A positive clone was obtained and cultivated at 37 °C. Following induction with 0.5 mM IPTG, the culture was cooled to 16 °C overnight to facilitate protein expression. The bacteria were then harvested by centrifugation and resuspended in a balanced buffer (200 mM sodium chloride, 25 mM Tris, 20 mM imidazole). The bacterial solution was homogenized twice under high pressure, followed by centrifugation at 17,000 rpm and 4 °C for 30 minutes. The resulting supernatant was collected and subjected to purification using a Ni affinity column. The eluted protein was cleaved with an appropriate amount of TEV protease and incubated at 16 °C for 2 hours. Subsequently, the protein solution was loaded onto a fresh Ni column to remove the tag protein and TEV enzyme, yielding the purified reEK1.

S protein-mediated cell-cell fusion and inhibition assays

Effector cells (293T/S/EGFP) bearing S protein on the surface through transfecting plasmid pAAV-IRES-S-EGFP. Calu-3 or Caco2 cells naturally expressing human ACE2 receptor were used as target cells as well as 293T/ACE2 cells. 293T cells transfected with plasmid pAAV-IRES-EGFP (293T/EGFP) were used as negative control effector cells. In the fusion kinetics assay, effector cells (293T/S/EGFP) were collected and added to target cells for co-incubation for 2, 4, 6, 8 and 24 hrs at 37 °C, respectively. In the fusion inhibition assay, the effector cells and target cells were co-incubated in the presence of EK1 or reEK1 for 4 hrs, and then pictures were taken under the fluorescence microscope (ThermoFisher EVOS M5000). The fused cell exhibits irregular shape and is at least 2-fold larger than the unfused cell. The fusion-inhibition percentage of cell-cell fusion was calculated using the following formula[4]: $[1 - (A - B)/(C - D)] \times 100\%$, where "A" represents the percentage of cell-cell fusion in the experimental group,

“C” represents the percentage of cell-cell fusion in the positive control group, and “B” represents the percentage of cell-cell fusion in the negative control group.

Western blot

Western blot was performed as previously described [5]. Effector cell samples after transfection for 36 hrs were collected and prepared to run an SDS-PAGE in 10% gels (Bio-Rad, Hercules, CA) and transferred onto PVDF membranes. Then membranes were blocked with BSA (5%) overnight, followed by incubation with SARS-CoV-2 poly-antibody (Sino Biological Inc., Cat: 40592-T62) for additional 2 hrs at room temperature with gentle agitation. Horseradish peroxidase (HRP)-conjugated polyclonal Goat anti-Rabbit IgG (1:5000) were used as secondary antibodies, respectively. Proteins were visualized using one-step ECL substrates (Meilunbio, Dalian, China). The S2/S protein were quantified by greyscale values by Image J.

Pseudovirus (PsV) infection and inhibition assays

PsVs were produced by co-transfecting plasmids carrying WT or mutant SARS-CoV-2 *S* gene with pNL4-3.Luc.R−E− vector. Medium was refreshed 12 hrs after the co-transfection. Then pseudovirus supernatant was collected 60 h post-transfection. The inhibitory efficacy of EK1 or reEK1 peptides against WT or mutant SARS-CoV-2 PsV was evaluated on Caco2 cells using a modified standard neutralization assay[6]. Briefly, a serially four-fold diluted peptide (60 μl) was incubated with PsV (60 μl) for ~30 min at 37℃. Then the mixture (100 μl) was transferred into Caco2 cells in a 96-well plate. After 12 hrs, the medium was refreshed. After an additional 48 hrs, luciferase value was tested by the Luciferase Assay System[6].

SDS-PAGE

Peptide EK1 and reEK1 was diluted in PBS, followed by mixing with loading buffer and then ran into an SDS-PAGE in 15.5% tricine-SDS-PAGE gel (Sangon, Shanghai, China). Gel was stained with Coomassie at room temperature for 30 min and then destained with destain solution until the protein bands are appeared with clean background.

Mouse infection assay

All mouse infection studies were performed in accordance with the animal experiment protocol approved by the Animal Experiment Committee of the School of Basic Medical Sciences, Fudan University (20210302-083). Female Tgtn (CAG-human ACE2-IRES-Luciferase) mice were purchased from were from Shanghai Model Organisms. Mice were challenged with 30,000 pfu SARS-CoV-2 BA.2 intranasally. 2 hrs later, mice were administered with reEK1 or vehicle (200 μg/mouse) through inhalation route once a day for 3 consecutive days times. Mice were euthanized on the 7th day-post-infection, and the lungs were collected for viral RNA level examination by RT-qPCR and pathological changes by HE stains. Viral RNA was extracted from supernatants of lung tissues using the EasyPureViral RNA Kit (TransGen, China) and then measured with a One-Step PrimeScript RT-PCR Kit (Takara, Japan). The sequences of probe and primers are as follows:

Probe: 5′-FAM-CCGTCTGCG GTATGTGGAAAGGTTATGG-BHQ1-3′

SARS-CoV-2-ORF1ab-F: CCCTGTGGGTTTTACACTTAA;

SARS-CoV-2-ORF1ab-R: ACGATTGTGCATCAGCTGA.

Statistical analyses

Statistical analyses were carried out using GraphPad Prism 8.0. Analyses of independent data were performed through Student's unpaired two-tailed *t*-test. *P* value less than 0.05 is significant; **P* < 0.05; ***P* < 0.01; ****P* <0.001; *****P*<0.0001. The concentration for half inhibition (IC50) was calculated by GraphPad Prism 8.0.

References

1 Xia, S. et al. A pan-coronavirus fusion inhibitor targeting the HR1 domain of human coronavirus spike. *Sci Adv* **5**, eaav4580 (2019).

2 Xia, S. et al. Inhibition of SARS-CoV-2 (previously 2019-nCoV) infection by a highly potent pan-coronavirus fusion inhibitor targeting its spike protein that harbors a high capacity to mediate membrane fusion. *Cell Res* **30**, 343-355 (2020).

3 Lan, Q. et al. Pan-coronavirus fusion inhibitors to combat COVID-19 and other emerging coronavirus infectious diseases. *J Med Virol* **95**, e28143 (2023).

4 Lu, L. et al. Structure-based discovery of Middle East respiratory syndrome coronavirus fusion inhibitor. *Nat Commun* **5**, 3067 (2014).

5 Xia, S. et al. Structure-based evidence for the enhanced transmissibility of the dominant SARS-CoV-2 B.1.1.7 variant (Alpha). *Cell Discov* **7**, 109 (2021).

6 Xia, S. et al. Structural and functional basis for pan-CoV fusion inhibitors against SARS-CoV-2 and its variants with preclinical evaluation. *Signal Transduct Target Ther* **6**, 288, (2021).

Supplement Figures

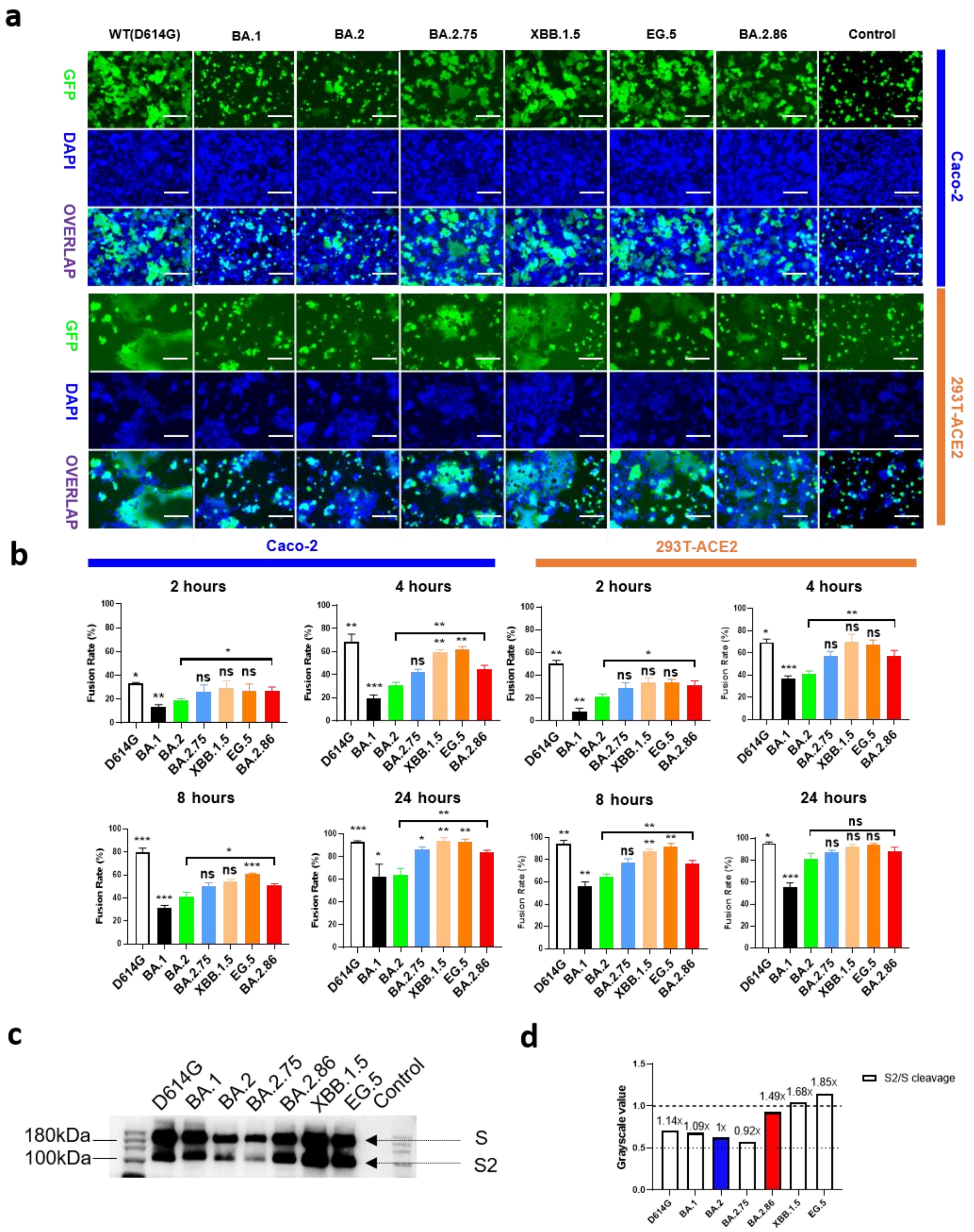

Figure S1. Membrane fusion kinetics driven by S proteins of BA.2.86 and other Omicron subvariants.

a. Representative images of cell-cell fusion mediated by 293T/WT(D614G)-S, BA.1-S, BA.2-S, BA.2.75-S, BA.2.86-S, XBB.1.5-S or EG.5-S cells on Caco-2 cells or 293T/ACE2 after coculture for 8 hrs. Scale bar = 150 µm.

b. Fusion rate mediated by WT(D614G)-S, BA.1-S, BA.2-S, BA.2.75-S, BA.2.86-S,

XBB.1.5-S and EG.5-S proteins on Caco2 or 293T-ACE2 cells after coculture for 2, 4, 8 and 24 hrs. As compared with the BA.2.86 group, asterisks indicate significant differences (*$P < 0.05$, **$P < 0.01$, ***$P < 0.001$).

c. Western blot analysis of S protein expression in effector cells.
d. Statistical analysis of the ratios of S2/S of BA.2.86-S and other Omicron variant-S proteins on effector cells.

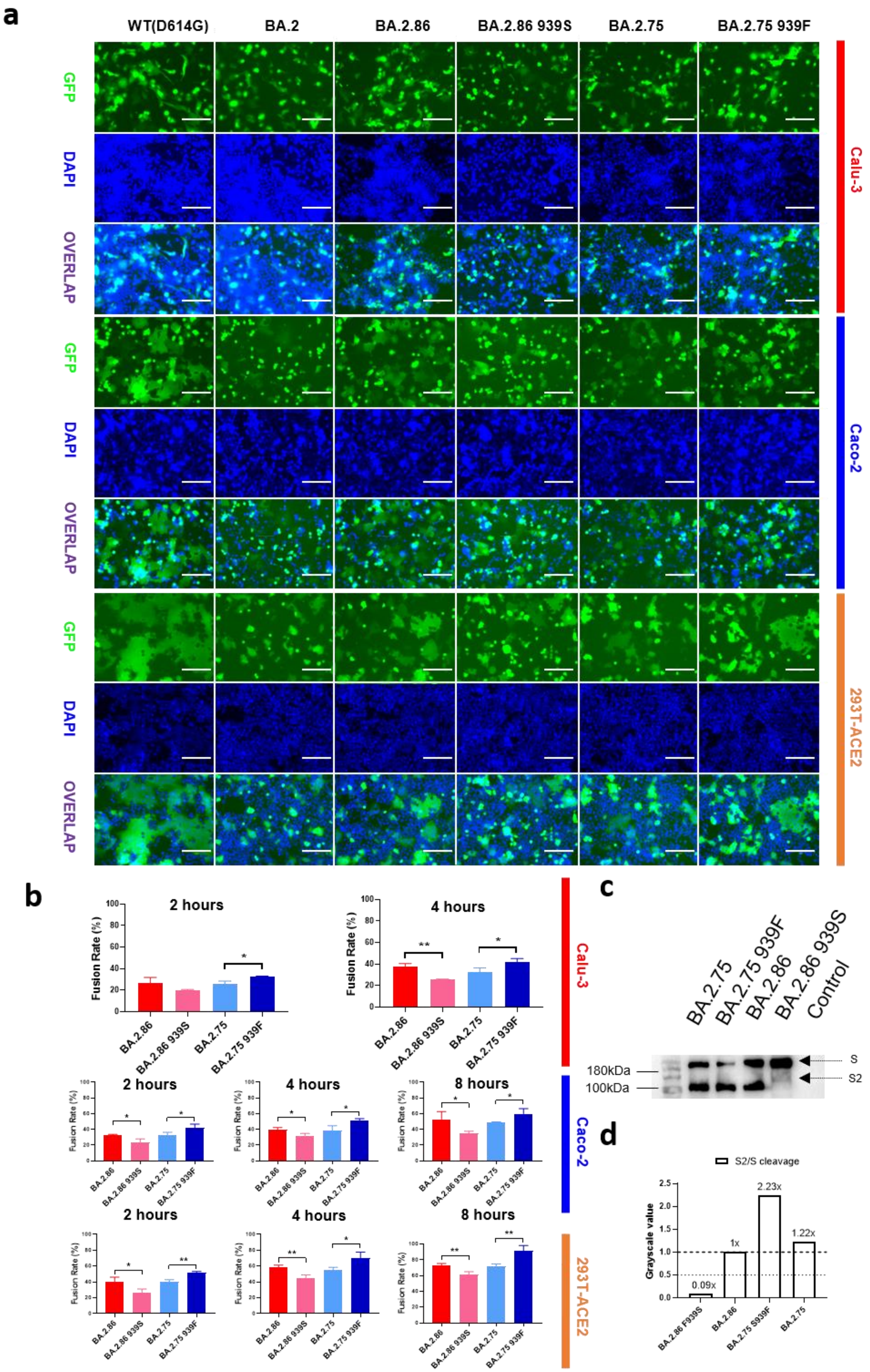
a
WT(D614G)
BA.2
BA.2.86
BA.2.86 939S
BA.2.75
BA.2.75 939F
GFP
DAPI
OVERLAP
Calu-3
Caco-2
293T-ACE2
b
2 hours
4 hours
8 hours
Fusion Rate (%)
BA.2.86
BA.2.86 939S
BA.2.75
BA.2.75 939F
c
BA.2.75
BA.2.75 939F
BA.2.86
BA.2.86 939S
Control
180kDa
100kDa
S
S2
d
S2/S cleavage
Grayscale value
0.09x
1x
2.23x
1.22x
BA.2.86 F939S
BA.2.86
BA.2.75 S939F
BA.2.75

Figure S2. Membrane fusion kinetics driven by S proteins of BA.2.86 and BA.2.75 with 939S/F mutation in HR1 region.

a. Representative images of cell-cell fusion mediated by WT(D614G)-S, BA.2-S, BA.2.75, BA.2.75-S-939F, BA.2.86-S, and BA.2.86-S-939S on Calu-3 cells, Caco-2 cells or 293T/ACE2, respectively, after coculture for 8 hrs. Scale bar = 150 μm.
b. Quantification of fusion rate mediated by WT(D614G)-S, BA.2-S, BA.2.75, BA.2.75-S-939F, BA.2.86-S, and BA.2.86-S-939S proteins on Calu-3 cells after coculture for 2 or 4 hrs, and that on Caco2 or 293T-ACE2 cells after coculture for 2, 4 and 8 hrs, respectively. As compared with the BA.2.75 or BA.2.86 group, asterisks indicate significant differences (*$P < 0.05$, **$P < 0.01$).
c. Western blot analysis of S protein expression and S protein cleavage in effector cells.
d. Quantification of the S2/S ratios of BA.2.75-S, BA.2.75-S-S939F, BA.2.86-S, BA.2.86-S-F939S on effector cells.

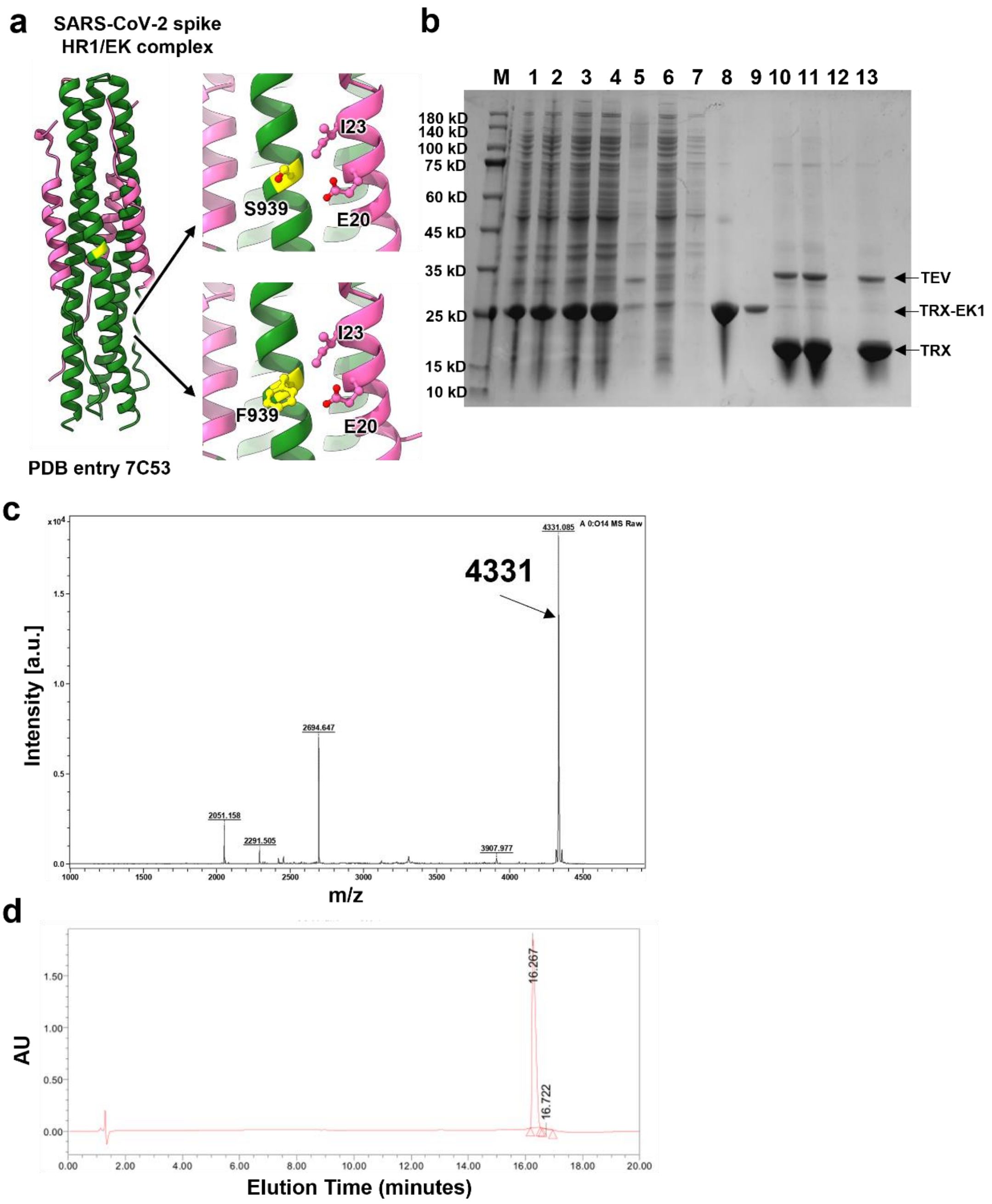

Figure S3. The S939F mutation in the HR1/EK1 complex and recombinant expression of the EK1 peptide.

a. The cartoon representation illustrates the structure of the WT HR1/EK1 complex (PDB entry 7G53), with the S939 residue highlighted in yellow. The predicted structure of the S939F mutant was obtained from the SWISS-MODEL server. A zoomed-in view showcases the local region surrounding S939 and F939, with important residues depicted as sticks and labeled accordingly.

b. Recombinant expression of the EK1 peptide was analyzed using SDS-PAGE. Due

to its low molecular weight, the band corresponding to the EK1 peptide was not detectable on the gel. The samples for each lane were as follows: Lane 1, fermentation liquor; Lane 2, first homogenized liquid; Lane 3, second homogenized liquid; Lane 4, centrifugal supernatant; Lane 5, centrifugal precipitation; Lane 6, flow-through of Ni column; Lane 7, impurity cleaning; Lane 8, elution by 250 mM imidazole solution; Lane 9, elution by 1 M imidazole solution; Lane 10, cleavage by TEV enzyme; Lane 11, buffer exchange; Lane 12, flow-through of reloading onto Ni column; Lane 13, elution of reloading.

c. The reEK1 solution was desalted using a ziptip C18 and then mixed with the CHCA substrate. Subsequently, the analysis was performed using a Matrix-Assisted Laser Desorption/Ionization Time-of-Flight Mass Spectrometer (MALDI-TOF/TOF) (UltraflextremeTM, Bruker, Germany) in reflection mode. The detected molecular weight of reEK1 was consistent with the theoretical molecular weight of EK1.

d. The purity of reEK1 was assessed using reversed-phase chromatography (RPC). The main peak (reEK1) exhibited a purity percentage of approximately 99% based on peak area normalization calculation method.

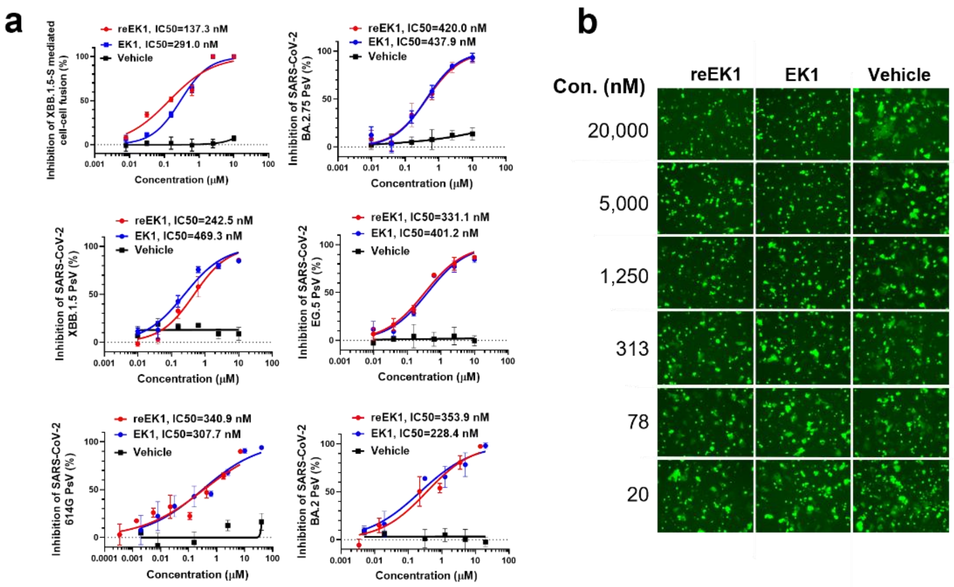

Figure S4. Inhibitory activity of reEK1 and EK1 against BA.2.86 and other Omicron subvariants on cell-cell fusion or pesudovirus (PsV) infection system.

a. Inhibitory activity of reEK1 and EK1 against XBB.1.5-S cell-cell fusion and WT(D614G), XBB.1.5, BA.2.75, EG.5, BA.2 PsVs infection.

b. Representative images of inhibition of reEK1 against BA.2.86-S mediated cell-cell fusion.
